# Cardiovascular and Respiratory Responses During Graded Exercise in Adolescents After Sport-Related Concussion

**DOI:** 10.1007/s40279-025-02301-7

**Published:** 2025-08-26

**Authors:** John J. Leddy, Mohammad N. Haider, Haley M. Chizuk, Muhammad S. Z. Nazir, Phillip Worts, Barry S. Willer, Blair D. Johnson

**Affiliations:** 1 UBMD Orthopaedics and Sports Medicine, SUNY Buffalo Jacobs School of Medicine and Biomedical Sciences, Buffalo, NY USA; 2 Tallahassee Orthopedic Clinic, Tallahassee, FL USA; 3 Department of Nutrition and Integrative Physiology, Florida State University Institute of Sports Sciences and Medicine, Tallahassee, FL USA; 4 Department of Psychiatry, SUNY Buffalo Jacobs School of Medicine and Biomedical Sciences, State University of New York at Buffalo, Buffalo, NY USA; 5 Department of Kinesiology, School of Public Health-Bloomington, Indiana University, Bloomington, IN USA

## Abstract

**Background:**

Symptom-limited exercise intolerance is a physiological sign of sport-related concussion. Possible etiologies include rest-induced aerobic deconditioning and/or impaired cardiopulmonary function.

**Objective:**

This study examined cardiovascular and respiratory function at rest and during progressive cycle ergometer exercise in adolescents within 10 days of sport-related concussion compared with non-concussed athletes.

**Methods:**

Concussed participants (*n* = 26, 15.4 ± 1.1 years, 54% male, 7.3 ± 1.8 days from injury) and control participants (*n* = 24, 15.8 ± 1.6 years, 58% male) performed the Buffalo Concussion Bike Test. Blood pressure, heart rate, stroke volume, cardiac output, respiratory rate, minute ventilation, oxygen consumption, and end-tidal CO_2_ were collected at rest and continuously during exercise.

**Results:**

Concussed participants exercised for 16.24 ± 5.6 min, experienced a greater than 2-point (on a 0–10 scale) exacerbation of their concussion symptoms at their final minute, and reported higher perceived exertion throughout exercise versus controls. Controls exercised for 25.08 ± 7.0 min up to voluntary exhaustion without reporting any concussion-like symptoms. Concussed participants’ cardiovascular and respiratory parameters did not differ at rest versus controls, but concussed participants had higher minute ventilation and their blood pressure plateaued at lower values during the first 10 min of exercise.

**Conclusions:**

No evidence of aerobic deconditioning was found within 10 days of injury. Our study found attenuated cardiopulmonary responses to progressive aerobic exercise, which may be a cause for exercise intolerance in concussed adolescent athletes. Additional research is warranted to determine if this may be related to altered autonomic nervous system regulation.

**Supplementary Information:**

The online version contains supplementary material available at 10.1007/s40279-025-02301-7.

## Key Points

**Table:** 

Exercise intolerance is common after sport-related concussion, but the exact reason is not known
Our experimental investigation on cardiovascular and respiratory responses to exercise in adolescents after sport-related concussion found no evidence of aerobic deconditioning
We found some differences in cardiopulmonary function that suggest exercise intolerance may be because of the inability of the heart to adequately increase blood pressure to meet the demands of the body during progressive aerobic exercise. Additional research is warranted to determine how altered autonomic nervous system regulation causes exercise intolerance within 10 days of concussion

## Introduction

Concussion-related exercise intolerance is defined as limited aerobic exercise performance because of a more-than-mild (i.e., > 2-point change from the pre-exercise value on a 0–10 point scale) exacerbation of concussion symptoms, including profound fatigue [1]. We have shown that exercise intolerance is common within 10 days of sport-related concussion (SRC) [2] and is a biomarker for physiological dysfunction that can be used to aid in concussion diagnosis [3]. The degree of exercise intolerance early after injury is also prognostic: a lower heart rate (HR) threshold at the more-than-mild symptom exacerbation point within the first 10 days of injury is associated with prolonged recovery [4]. Return of exercise tolerance also helps to establish physiological recovery from SRC [5]. The etiology of concussion-related exercise intolerance is not completely understood. Potential mechanisms include aerobic deconditioning from rest and not participating in exercise or sport, impaired cardiopulmonary function, and/or altered cerebral blood flow (CBF) regulation [6, 7].

The body’s hemodynamic and respiratory functions in response to exercise are controlled by the sympathetic and parasympathetic branches of the autonomic nervous system (ANS) [8, 9]. Some athletes with SRC show greater sympathetic nervous system activation when compared with controls, exemplified by higher HRs at rest and during cognitive or physical activity [10–12], whereas other studies have shown lower HRs in acutely concussed athletes at rest and during graded exercise testing when compared with controls [13]. These seemingly contradictory responses suggest that concussed patients are not able to appropriately regulate cardiac autonomic activity [13]. The heart and arteries respond to autonomic efferent activity to regulate blood pressure (BP) and to maintain tissue perfusion [14]. Although cardiac output (CO), the product of HR and stroke volume (SV), [15] can be impaired after moderate and severe traumatic brain injury [19], there is little information on CO after concussion. Another possibility is that rest-induced aerobic deconditioning limits exercise performance after concussion, but there is a lack of evidence to support this theory [16, 17].

The current case–control study examines cardiopulmonary function at rest and during the first 10 min of progressive exercise on the Buffalo Concussion Bike Test (BCBT) [18] within 10 days of SRC in adolescent athletes compared to non-concussed controls. Our aim was to determine whether cardiovascular and/or respiratory responses to exercise differed between concussed and healthy adolescent athletes and to identify evidence of aerobic deconditioning in concussed adolescents within 10 days of injury. We hypothesized that there would be differences in the rate of change of cardiopulmonary responses during exercise in concussed versus non-concussed athletes, but that there would be no evidence of aerobic deconditioning in the first 10 days after SRC. Our null hypothesis was that there would not be any differences.

## Methods

### Study Design

This prospective case–control study was approved by the University at Buffalo Institutional Review Board (STUDY00000092) and the procedures used in this study adhere to the tenets of the Declaration of Helsinki. Concussed adolescents were recruited from three university-affiliated sports medicine clinics between September 2016 and March 2020 and were diagnosed by experienced physicians who followed international guidelines [19]. Healthy athletic controls were recruited from local high schools. Research assistants explained the study, screened for eligibility, and obtained written consent in a Health Insurance Portability and Accountability Act-compliant setting. Parental consent and participant assent were obtained for all minors (age 13–17 years).

### Participants and Concussion Diagnosis

Inclusion criteria for concussed adolescents included: (1) age 13–18 years; (2) any race, ethnicity, sex, or gender; (3) sustained physician-diagnosed concussion from sport-related activity within 10 days of the first visit; (4) demonstrated exercise intolerance on graded exertion testing [3]; and (5) reported a symptom severity score of ≥ 7 on the Post-Concussion Symptom Scale (maximum = 132). [20] The Post-Concussion Symptom Scale is a reliable measure with an internal consistency of 0.93. [21] Exclusion criteria included: (1) current or prior head injury more severe than a concussion; (2) inability to exercise (e.g., orthopedic injury or increased cardiac risk); (3) active substance abuse/dependence; (4) more than three previous diagnosed concussions or still experiencing symptoms from prior concussion; or (5) currently on medication that affects autonomic function (e.g., beta-blockers, calcium blockers, stimulants, or mood stabilizers). Inclusion criteria for healthy adolescents were: (1) age 13–18 years; (2) any race, ethnicity, sex, or gender; (3) not currently experiencing a concussion and at least 1 year after having recovered from the most recent concussion; and (4) currently participating in at least one organized sport [22]. Exclusion criteria for healthy adolescents were identical to those for participants with concussion.

### Experimental Protocol and Measurements

The study was conducted in an exercise physiology laboratory within the sports medicine clinic building within 10 days of concussion. Participants not tested on the day of diagnosis were instructed that they could do light activity (e.g., walking and activities of daily living) but not exercise prior to the study visit. The room was approximately 400 square feet in area, and the temperature and humidity were controlled. Participants were asked to abstain from caffeine, alcohol, nicotine (vapes), and vigorous physical activity on the day of physiological testing and to fast for at least 3 h before the visit. After instrumentation, participants were instructed to sit on a recumbent cycle ergometer and rest for 10 min prior to a 1-min collection of baseline measurements. Vital signs were inspected for 10 min at seated rest to confirm they were stable prior to the 1-min pre-exercise measurement (1 min was used to be consistent with the 1 min of exercise data collected at the end of each stage on the bike). Participants exercised according to the BCBT protocol [18], a progressive cycle exercise test that is metabolically equivalent to the Buffalo Concussion Treadmill Test. Participants pedaled at a 60-rpm cadence consistently while the resistance (Watts) was increased every 2 min. The resistance at each stage was based upon the participant’s weight using the American College of Sports Medicine metabolic equations for cycle ergometers [23]. The metabolic equivalents of task for seated rest were 1; for minutes 0–2, 3.74 (Stage 1); for minutes 2–4, 4.24 (Stage 2); for minutes 4–6, 4.73 (Stage 3); for minutes 6–8, 5.22 (Stage 4); and for minutes 8–10, 5.72 (Stage 5). Complete details on the BCBT protocol have been published [18]. Participants were asked to report their concussion symptoms on a 0–10 visual analog scale and their rating of perceived exertion (RPE, 6–20 Borg scale) at the end of each BCBT stage (i.e., every 2 min). Participants exercised until a more-than-mild symptom exacerbation (defined as an increase of > 2 points on the visual analog scale when compared with the pre-exercise resting value) or to voluntary exhaustion (defined as an RPE ≥ 18).

#### Data Acquisition

Data were continuously recorded at 2000 Hz using a data acquisition system (MP150; BIOPAC Systems Inc., Goleta, CA, USA). Mean values for each participant were calculated offline over the last minute of each exercise stage (AcqKnowledge 5.0).

#### Cardiovascular Variables

Beat-to-beat BP and HR were obtained using finger photoplethysmography (Nexfin; BMEYE, Amsterdam, Netherlands). The Heart Reference System was placed at the level of the heart and the Physiocal™ vascular unloading algorithm was disabled after baseline measurements were acquired. Stroke volume was estimated using the arterial pressure waveform from Modelflow [24], and CO was calculated as the product of HR and SV. Systemic vascular resistance was estimated by dividing mean arterial pressure (MAP) by CO. Systolic BP and diastolic BP (DBP) were obtained directly from the device. HR was also collected using a three-lead electrocardiogram to confirm that the photoplethysmograph’s HR recording was accurate. We did not, however, measure BP manually to confirm the accuracy of the photoplethysmograph’s BP recording. However, the Nexfin has been reported to provide accurate readings [25].

#### Respiratory Variables

Participants breathed through a one-way valve (Hans Rudolph, Kansas City, KC, USA) with a nose clip. The fractions of O_2_ and CO_2_ in expired air (FeO_2_, FeCO_2_) were analyzed from a 5-L mixing chamber using calibrated gas analyzers (BIOPAC Systems Inc.). Tidal volume and respiratory rate (RR) were obtained using a pneumotach (Pneumotach Transducer; BIOPAC Systems Inc.). Minute ventilation (MV) was calculated as the product of tidal volume and RR and converted to L/min. Capnography was used to measure end-tidal CO_2_ (EtCO_2_) [Respsense, Nonin Medical] at the mouth to estimate arterial CO_2_. Volume of oxygen consumed (*V*O_2_) and volume of carbon dioxide consumed (*V*CO_2_) were calculated with the Haldane transformation [26], and the respiratory exchange ratio was calculated by *V*CO_2_/*V*O_2_.

### Statistical Analysis

Participant demographics and resting physiological parameters were compared at baseline using independent sample *t*-tests and *χ*^2^ tests. Mean values were plotted over Watts per stage during the BCBT. Similar graphs plotted against time are provided in the Electronic Supplementary Material (ESM). Mixed model (fixed and random effects) linear regressions were used to compare groups, with the fixed main effects of power (Watts, continuous) and concussion (yes/no) and the fixed interaction term of concussion and Watts as predictors for each physiological variable. An autoregressive covariance pattern was identified, and random effects were participant specific. Only the first five stages (i.e., 10 min) of the BCBT were compared over time as all concussed participants reached this stage and experienced symptom exacerbation at subsequent stages. This is explained in more detail on p. 1 of the ESM. Physiological parameters were also compared at the final stage of exercise immediately before each participant ended the test. The final minute analysis is confounded by the total time spent exercising, which was shorter for concussed participants. A *p*-value of < 0.05 was considered statistically significant, and all analyses were performed using SPSS Version 29 (IBM Corp, Armonk, NY, USA) [27]. Graphs were made using Prism GraphPad Version 10 (see ESM).

## Results

### Sample Inclusion and Demographics

Ninety-nine adolescents were eligible and 33 were interested in participating. Sixty-six were not interested in participating in research and did not want to know more details. Of the 33 who were interested, six were not able to schedule a time to come for a 4-h research visit within 10 days of injury and did not participate. One participant who performed the research assessment was excluded because of missing physiological data because of technician error. Twenty-five non-concussed athletes contacted us and consented to perform the study, but one was not able to schedule a visit. Hence, the study sample consisted of 26 concussed participants (15.4 ± 1.1 years, 54% male, 7.3 ± 1.8 days since injury) and 24 healthy controls (15.8 ± 1.6 years, 58% male). Figure e1 in the ESM presents the sample inclusion flow chart and Table 1 presents groupwise demographics. Three controls (12.5%) and 19 (73%) concussed participants had a prior concussion greater than 1 year ago, which was significantly different.

**Fig. 1:**
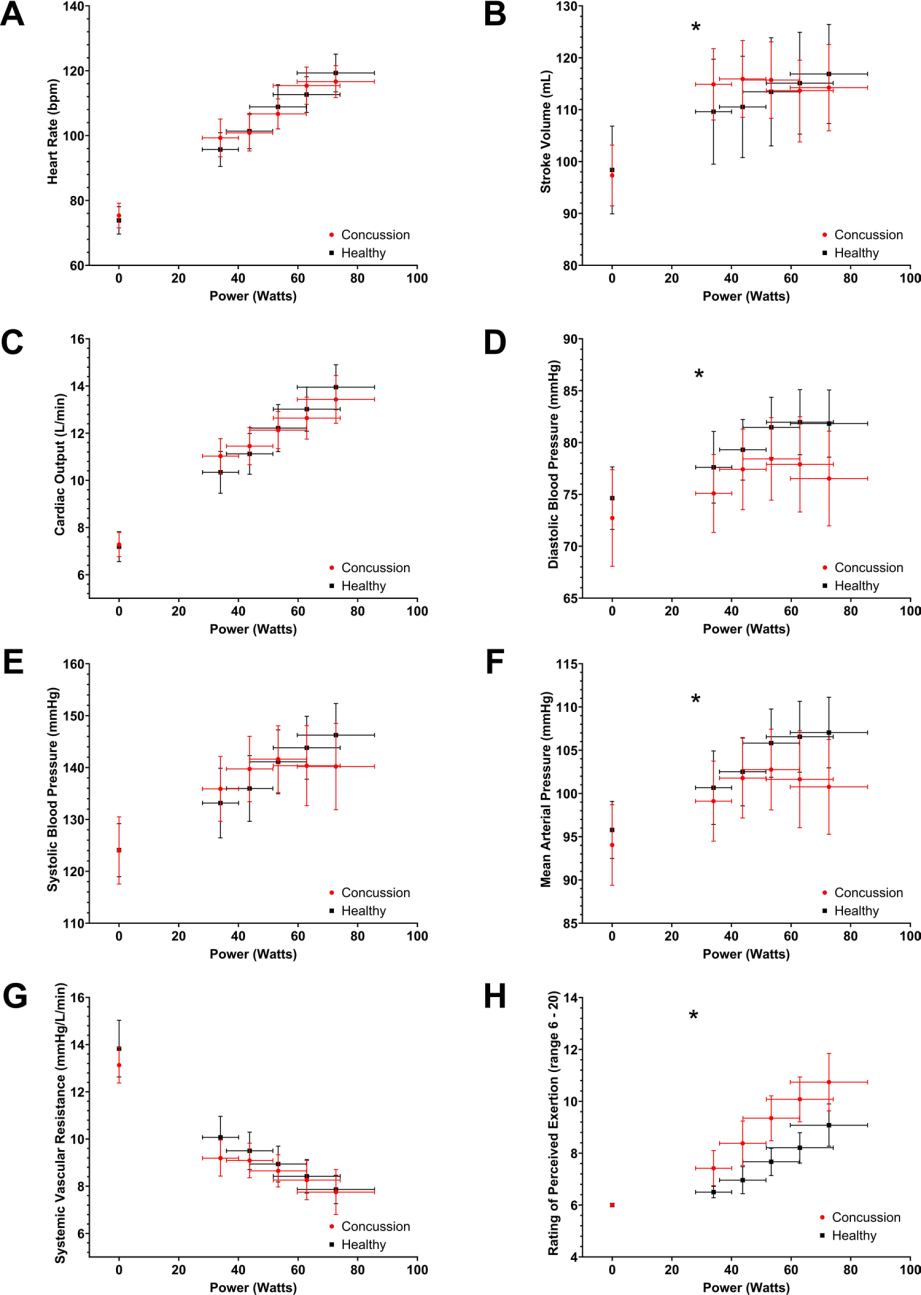
Mean (with 95% confidence interval) heart rate (**A**), stroke volume (**B**), cardiac output (**C**), diastolic blood pressure (**D**), systolic blood pressure (**E**), mean arterial pressure (**F**), systemic vascular resistance (**G**), and rating of perceived exertion (**H**) plotted against Watts during the first five stages of the Buffalo Concussion Bike Test. *p < 0.05 for the interaction term of Concussion*Watts

**Table 1:** Groupwise demographics and clinical characteristics

Variable	Concussion group	Healthy group	*p*-Value
Sample size, *n*	26	24	–
Age, mean (95% CI), years	15.42 (14.96, 15.88)	15.83 (15.18, 16.49)	0.289
Sex, *n* (%) male	14 (53.9)	14 (58.3)	0.749
Previous concussion, *n* (%)			
0	7 (26.9)	21 (87.5)	** < 0.001**
1	15 (57.7)	1 (4.2)	
2	2 (7.7)	1 (4.2)	
3	2 (7.7)	1 (4.2)	
Height, mean (95% CI), m	1.69 (1.64, 1.74)	1.70 (1.67, 1.74)	0.630
Weight, mean (95% CI), kg	69.34 (64.30, 74.38)	66.72 (61.66, 71.79)	0.454
Time since injury, days	7.27 (6.54, 8.00)	-	-
Time to recovery, days	28.12 (19.52, 36.71)	–	–
PPCS incidence, *n* (%)	9 (34.6)	–	–
Symptom severity total, mean (95% CI) [max = 132]	35.32 (26.11, 44.53)	0.81 (0.00, 3.00)	** < 0.001**
Symptom severity pre-injury, mean (95% CI) [max = 132]	2.86 (0.68, 5.05)	–	–
Sport of injury/primary sport	Baseball, *n* = 1 Basketball, *n* = 1 Dodgeball, *n* = 2 Football, *n* = 6 Ice hockey, n = 8 Lacrosse, *n* = 3 Soccer, *n* = 4 Volleyball, *n* = 1	Baseball, *n* = 2 Softball, *n* = 1 Dance, *n* = 1 Field hockey, *n* = 3 Football, *n* = 5 Ice hockey, *n* = 4 Lacrosse, *n* = 4 Horse riding, *n* = 1 Soccer, *n* = 1 Track, *n* = 1 Volleyball, *n* = 1	–

Bolded values indicate a significant finding

*CI* confidence interval, *max* maximum, *PPCS* persisting post-concussive symptom

### Comparison of Cardiovascular and Respiratory Measures at Rest

Table 2 presents mean cardiovascular and respiratory variables at rest. No significant differences were observed in any parameter. Medians and interquartile ranges for each variable are provided in Table e4 in the ESM.

**Table 2:** Groupwise cardiovascular and respiratory variables at rest

Variable	Concussion group	Healthy group	*p*-Value
Cardiovascular			
Heart rate (beats/min)	75.36 (71.56, 79.16)	73.85 (69.64, 78.06)	0.509
Stroke volume (mL)	97.33 (91.48, 103.18)	98.38 (89.92, 106.84)	0.782
Cardiac output (L/min)	7.28 (6.77, 7.79)	7.19 (6.55, 7.82)	0.882
Diastolic blood pressure (mmHg)	72.72 (68.07, 77.38)	74.64 (71.63, 77.65)	0.233
Systolic blood pressure (mmHg)	124.03 (117.55, 130.50)	124.08 (118.96, 129.19)	0.431
Mean arterial pressure (mmHg)	94.05 (89.39, 98.71)	95.79 (92.48, 99.09)	0.180
Systemic vascular resistance (mmHg/L/min)	13.13 (12.37, 13.89)	13.83 (12.63, 15.03)	0.242
Respiratory			
Respiratory rate (breaths/min)	18.94 (17.58, 20.30)	18.20 (1588, 20.53)	0.827
Tidal volume (mL)	431.82 (365.15, 498.50)	450.23 (392.98, 507.47)	0.927
Minute ventilation (L/min)	8.325 (7.365, 9.275)	8.287 (7.145, 9.415)	0.726
*V*O_2_ (mL/min/kg)	3.57 (2.87, 4.28)	3.74 (3.10, 4.39)	0.841
*V*CO_2_ (mL/min/kg)	2.09 (1.35, 2.84)	1.86 (1.38, 2.34)	0.548
RER (ratio)	0.53 (0.39, 0.67)	0.51 (0.41, 0.61)	0.814
EtCO_2_ (mmHg)	35.14 (33.14, 37.15)	32.65 (29.55, 35.75)	0.307

Values are provided as means with 95% confidence intervals; median with interquartile ranges are provided in Table e4 in the ESM

*EtCO*_*2*_ end-tidal CO_2_, *min* minute, *RER* respiratory exchange ratio, *VCO*_*2*_ volume of carbon dioxide consumption, *VO*_*2*_ volume of oxygen consumption

### Comparison of Cardiovascular Measures During the First 10 Min of Exercise

Figure 1 presents cardiovascular variables and RPE plotted as a function of Watts. The same variables plotted against time are provided in Fig. e2 of the ESM. Table e1 of the ESM provides mean values for variables in Fig. 1.

**Fig. 2:**
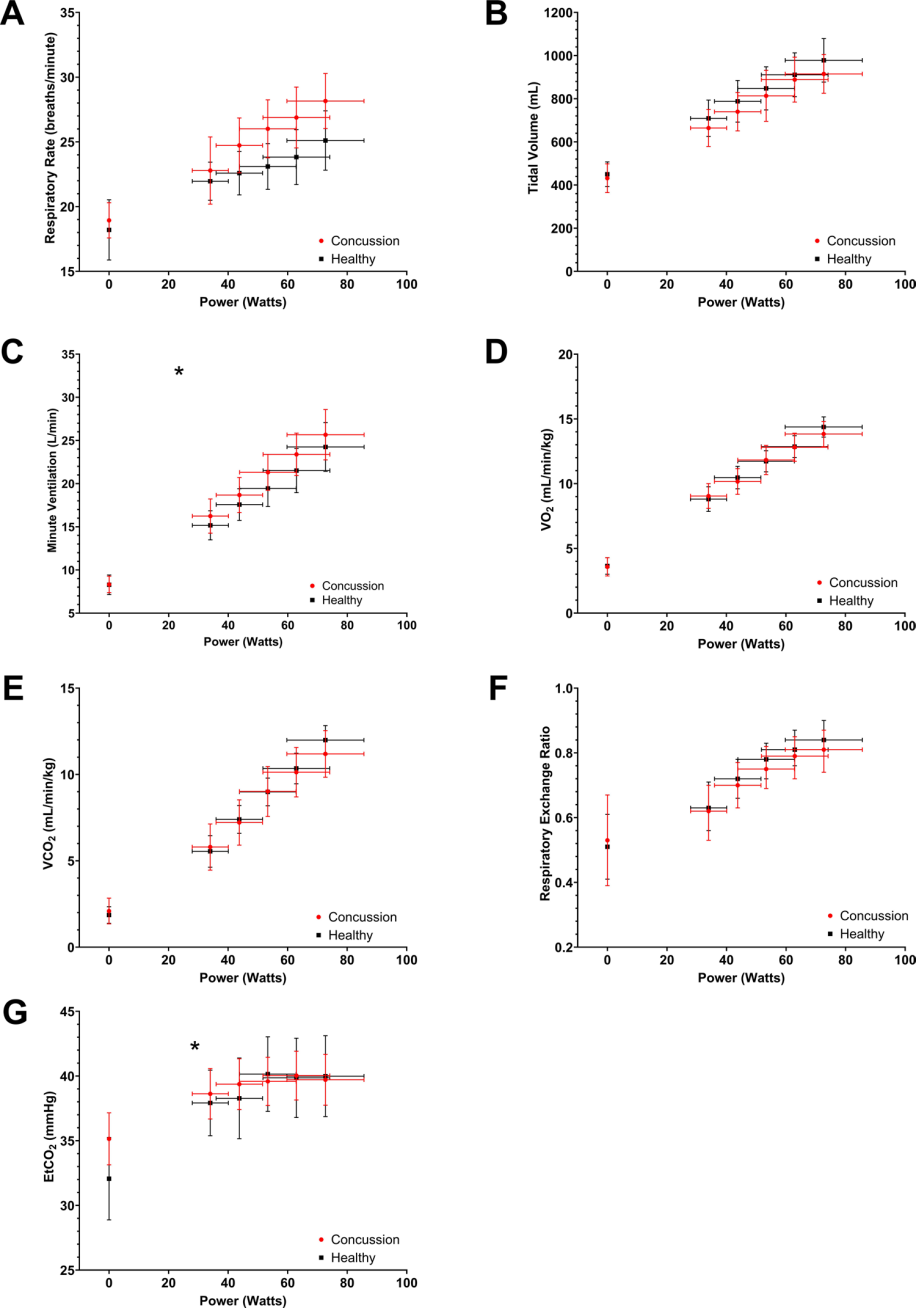
Mean (with 95% confidence interval) respiratory rate (**A**), tidal volume (**B**), minute ventilation (**C**), volume of oxygen consumed [*V*O_2_] (**D**), volume of carbon dioxide consumed [*V*CO_2_] (**E**), respiratory exchange ratio (**F**), and end-tidal carbon dioxide [EtCO_2_] (**G**) plotted against Watts during the first five stages of the Buffalo Concussion Bike Test. *p < 0.05 for the interaction term of Concussion*Watts

Table 3 summarizes the linear regressions for cardiovascular variables and RPE against Watts. The parameter estimate of the interaction term is the mean difference in rate of change per Watts between groups and is our primary comparison. All cardiovascular variables and self-reported exertion changed with increasing Watts (*p* < 0.001 for all) and there were significant differences in the rate of change between groups for SV, DBP, MAP, and RPE. Concussed participants had a greater increase in SV and RPE and smaller increase in DBP and MAP during exercise when compared with controls.

**Table 3:** Summary of linear regression of cardiovascular variables and RPE against Watts

Variable	Concussion rate of change^a^	Healthy rate of change	Mean difference in change^b^	*p*-Value
Heart rate (beats/min)	0.677 (0.601, 0.753)	0.684 (0.631, 0.738)	0.022 (− 0.071, 0.115)	0.640
Stroke volume (mL)	0.434 (0.376, 0.492)	0.296 (0.253, 0.339)	0.183 (0.112, 0.253)	** < 0.001**
Cardiac output (L/min)	0.096 (0.088, 0.104)	0.093 (0.088, 0.099)	0.003 (− 0.007, 0.013)	0.515
Diastolic blood pressure (mmHg)	0.051 (0.019, 0.084)	0.139 (0.100, 0.177)	− 0.105 (− 0.157, − 0.052)	** < 0.001**
Systolic blood pressure (mmHg)	0.293 (0.249, 0.337)	0.313 (0.221, 0.406)	− 0.063 (− 0.129, 0.003)	0.063
Mean arterial pressure (mmHg)	0.114 (0.080, 0.147)	0.204 (0.161, 0.248)	− 0.100 (− 0.156, − 0.045)	** < 0.001**
Systemic vascular resistance (mmHg/L/min)	− 0.074 (− 0.086, − 0.062)	− 0.093 (− 0.103, − 0.083)	0.008 (− 0.009, 0.026)	0.333
Rating of perceived exertion	0.060 (0.048, 0.073)	0.042 (0.035, 0.050)	0.016 (0.002, 0.030)	**0.021**

Bolded values indicate a significant finding

^a^Rates of change are upon 1 Watt

^b^This is the parameter estimate of the interaction term of Concussion X Watts and can be interpreted as the difference between the rate of change of each physiological parameter upon 1 Watt

### Comparison of Respiratory Measures During the First 10 Min of Exercise

Figure 2 presents respiratory variables plotted against Watts. The same variables plotted against time are provided in Fig. e4 of the ESM. Table e2 of the ESM provides mean values for variables in Fig. 2.

**Fig. 3:**
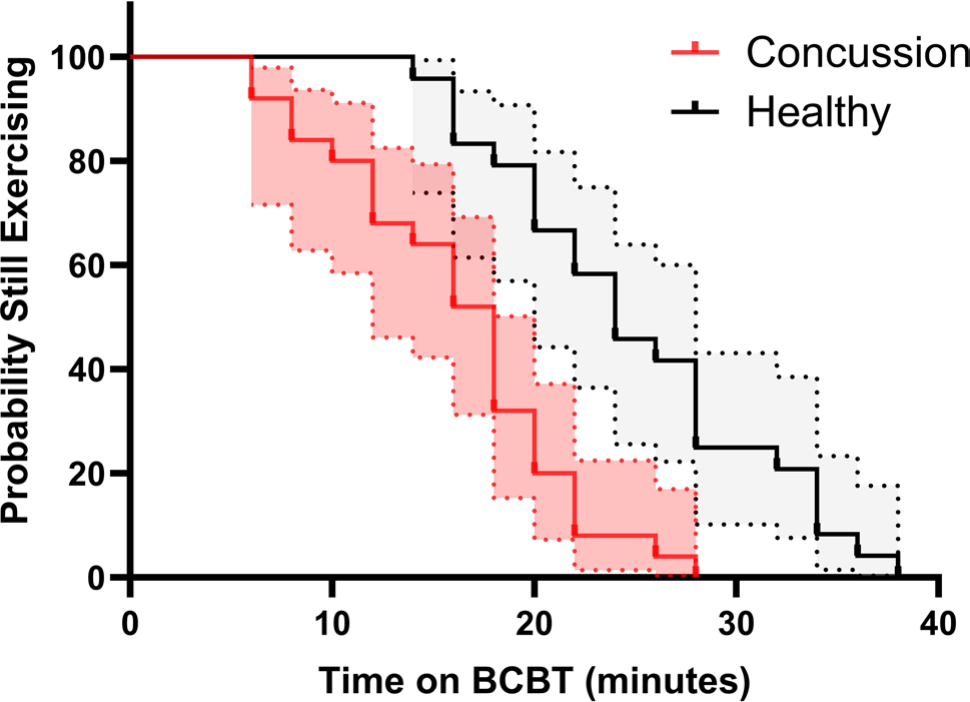
Total exercise duration on the Buffalo Concussion Bike Test (BCBT)

Table 4 summarizes the linear regressions for respiratory variables against Watts. All respiratory variables changed with increasing Watts (*p* < 0.001 for all). Significant differences were observed in the rate of change of MV and EtCO_2_ during exercise, with concussed participants having a greater increase in MV and a lesser increase in EtCO_2_.

**Table 4:** Summary of linear regression of respiratory parameters against Watts

Parameter	Concussion rate of change^a^	Healthy rate of change	Mean difference in change^b^	*p*-Value
Respiratory rate (breaths/min)	0.132 (0.099, 0.165)	0.097 (0.069, 0.124)	0.036 (− 0.007, 0.079)	0.096
Tidal volume (mL)	7.625 (6.369, 8.881)	7.511 (6.339, 8.682)	− 0.568 (− 1.727, 0.591)	0.335
Minute ventilation (L/min)	0.248 (0.229, 0.266)	0.215 (0.200, 0.231)	0.030 (0.005, 0.055)	**0.020**
*V*O_2_ (mL/min/kg)	0.144 (0.131, 0.157)	0.147 (0.136, 0.157)	− 0.002 (− 0.018, 0.015)	0.834
*V*CO_2_ (mL/min/kg)	0.117 (0.103, 0.132)	0.125 (0.114, 0.137)	− 0.010 (− 0.033, 0.013)	0.397
RER (ratio)	0.004 (0.003, 0.005)	0.004 (0.003, 0.005)	0.001 (− 0.001, 0.002)	0.428
EtCO_2_ (mmHg)	0.082 (0.065, 0.099)	0.126 (0.091, 0.161)	− 0.045 (− 0.007, − 0.084)	**0.022**

Bolded values indicate a significant finding

*EtCO*_*2*_ end-tidal CO_2_, *min* minute, *RER* respiratory exchange ratio, *VCO*_*2*_ volume of carbon dioxide consumption, *VO*_*2*_ volume of oxygen consumption

^a^Rates of change are upon 1 Watt

^b^This is the parameter estimate of the interaction term of Concussion X Watts and can be interpreted as the difference between the rate of change of each physiological parameter upon 1 Watt

### Comparison of Cardiovascular and Respiratory Measures During the Final Minute of Exercise

Concussed participants exercised for a mean duration of 16.24 ± 5.9 min (8.12 ± 3.0 stages) with a minimum of 10 min (five stages). Control participants exercised for 25.08 ± 7.0 min (12.54 ± 3.5 stages, *p* < 0.001) without reporting any concussion-like symptoms. A Kaplan–Meier survival graph of total time on the BCBT is presented in Fig. 3. A log-rank test comparing survival function between groups was statistically significant (*p* < 0.001). Table e3 in the ESM compares the final minute of exercise between concussed participants (16 min on average) and controls (25 min on average). Controls achieved higher values in RPE, maximum power output, HR, CO, MV, *V*O_2_, and *V*CO_2_ versus adolescents with concussion.

## Discussion

This extensive evaluation of cardiovascular and respiratory function informs our understanding of the etiology of exercise intolerance in adolescents early after SRC. Our main findings are that there were no cardiopulmonary differences between concussed and healthy adolescents at rest. During progressive cycle exercise, however, concussed participants experienced exercise intolerance in association with altered perceived exertion, SV, DBP, MAP, MV, and EtCO_2_ versus controls at equivalent intensities. Concussed participants did not exercise for as long as controls, stopping the exercise test at a lower RPE because of an exacerbation of concussion symptoms. During the first 10 min of the exercise test, however, concussed participants reported greater RPE than controls at each stage. This is consistent with our prior report that adolescent athletes after SRC experience a higher perceived exertion than healthy athletes during exercise at equivalent intensities [1, 13]. Several differences were observed during the final minute of exercise (16 min in the concussion group and 25 min in the control group), with healthy participants exercising to a higher intensity, a higher perceived exertion, and higher HR, CO, MV, *V*O_2_, and *V*CO_2_. Physiological differences during the final minute were expected because healthy participants exercised for a significantly longer period and were not limited by worsening of concussion-like symptoms.

### Cardiopulmonary Responses to Exercise

Concussed participants had a greater increase in SV but limited increases in DBP and MAP with increasing exercise intensity versus controls. Examining the SV graph (Fig. 1B) shows that there was a large increase in SV initially that plateaued at a lower value by Stage 5 of the BCBT. Diastolic BP (Fig. 1D) and MAP (Fig. 1F), however, initially increased normally but plateaued at approximately 10 mmHg lower than healthy controls. A similar pattern was also seen for SBP, but it did not reach statistical significance. These are the very factors that limit performance in healthy people during a progressive exercise test [28, 29]. The inability of the heart to further increase cardiac output as exercise intensity increases blunts the increase in MAP necessary to maintain adequate blood flow and oxygen delivery to meet the increasing skeletal muscle demands during an exercise test. Thus, these data suggest that limitations in cardiac function to maintain adequate BP may be the reason for reduced exercise tolerance after SRC. We did not perform direct quantification of the ANS (e.g., the Ewing test [30]) or of cardiac function (e.g., echocardiogram), so we cannot say with certainty that exercise intolerance was due to altered cardiac autonomic regulation versus cardiac muscle dysfunction. However, the most plausible mechanism for the reduced cardiac response in concussed participants is altered cardiac autonomic function, which has been well documented after concussion [31]. In support of this, our prior studies show that recently concussed athletes have attenuated sympathetically mediated BP responses to sympathoexcitatory stimuli [32, 33]. Additionally, improved cardiac autonomic function may be one reason for the efficacy of early sub-threshold aerobic exercise for facilitating recovery from SRC [34, 35], but further research is warranted to confirm this hypothesis.

With respect to respiratory parameters, concussed adolescents increased MV at a higher rate than healthy adolescents, which was most likely due to a higher RR rather than a change in tidal volume. Increased breathing rates are difficult to interpret in this context because concussed participants were symptomatic and likely experiencing some level of discomfort. Additionally, concussed participants may have felt more anxious because they were acutely symptomatic, which is a common symptom after SRC. Both pain and anxiety are independent confounders of increased breathing rates [36], so we are unsure if the differences we observed are due to autonomic dysfunction or due to external stressors. Concussed participants also had a lower rate of rise in EtCO_2_ (Fig. 2G) and a slightly higher resting EtCO_2_ that plateaued at the same partial pressures as in healthy adolescents. The slightly higher resting EtCO_2_, albeit non-significant, with no differences in resting RR in concussed adolescents may be due to altered central chemosensitivity [37]. In a prior study, we found attenuated central chemosensitivity during aerobic exercise (i.e., a reduced ventilatory response to hypercapnia) [38] in female college athletes with persisting concussion symptoms when compared with controls [39]. In the present study, however, we found a greater MV increase in concussed adolescent athletes with no differences in *V*O_2_, *V*CO_2_, or the respiratory exchange ratio, which suggests improved central chemosensitivity [40]. The different results likely reflect different study samples as the previous study included college-aged female individuals who were greater than 6 weeks from injury, and prolonged inactivity and deconditioning may have adversely affected their central chemosensitivity [41].

### Aerobic Deconditioning

There has been speculation in the literature [16, 17] that aerobic deconditioning due to rest and reduced physical activity may cause exercise intolerance in athletes with persisting symptoms after concussion. The physiological responses that define aerobic deconditioning include increased HR at rest and during exercise, increased resting BP, and lower resting SV [42, 43]. We did not observe any of these changes in our concussed athletes who were experiencing symptom-limited exercise intolerance within 10 days of injury. This is consistent with the exercise physiology literature, as the physiological changes related to aerobic detraining typically do not appear until after 12–14 days of rest, and athletes removed from activity typically regress toward pre-training status only after 4 or more weeks of stopping training [44–46]. This is well beyond the mean time of the first physiological testing of our concussed athletes (7 days from injury). Although concussed adolescents exercised for a shorter duration of time, oxygen consumption (i.e., *V*O_2_) at rest and during comparable exercise intensities did not differ between groups, indicating that our participants were well matched on the initial aerobic fitness level. Therefore, we suspect that it is highly unlikely that aerobic deconditioning caused exercise intolerance in adolescent athletes within 10 days of SRC. While continuous supine bed rest for 2 weeks can induce aerobic deconditioning [47], this was not applicable to our participants as concussed adolescents were instructed to not perform strict bed rest. Participants attended school, and while they were advised to avoid exercise and sport, engaging in light physical activity (walking, light household chores) was safe [48].

### Other Causes of Exercise Intolerance

Another potential cause for symptom exacerbation during physical activity after SRC is dysregulation of CBF [49]. We previously found that CBF velocity increased during a central hypervolemic challenge in concussed athletes [50], and other investigators have identified altered cerebral vascular function following concussion [51, 52]. During exercise, the ANS controls CBF by several [53] autoregulatory mechanisms to maintain constant cerebral perfusion pressure (CPP, i.e., the BP inside the brain). The two major autoregulatory mechanisms are (1) cerebral autoregulation (which responds to changes in systemic BP) and (2) cerebral vasoreactivity (which responds to changes in vasoactive chemicals, mainly CO_2_) [49]. Rotational forces applied to the upper cervical spine during concussive injury may damage these ANS centers within the brainstem and their connections to other centers [54, 55]. Neuroimaging research has identified brainstem changes using diffusion tensor imaging [56], and physiological research has demonstrated altered sympathetic [33] and parasympathetic [32] responses to stimuli after concussion. These data suggest that abnormal CBF regulation may contribute to exercise intolerance. We measured CBF velocity in a subset of the current study sample and followed them to clinical recovery. The results will be published in a separate article.

### Limitations

A major limitation is that we did not perform an a priori sample size calculation and were likely underpowered to assess rates of change for all 15 physiological parameters. Therefore, our results are not definitive and require validation using adequately powered samples. A second major limitation is that we did not control our analysis for participant sex. There are sex differences in exercise physiology. Including participant sex as a single main effect in regression models does not adequately account for sex differences and we were underpowered to include it as an interaction term with the other main effects (Watts and group). Therefore, we recommend future studies perform a sample size estimation for male and female individuals and analyze their data independently. Another limitation is that we did not include concussed participants who were not experiencing symptom-limited exercise intolerance. We therefore cannot unequivocally state that the differences we measured are the source for exercise intolerance or whether these changes may occur in all adolescents with SRC. An exploratory meta-analysis showed that most (approximately 95%) adolescent athletes experience some degree of exercise intolerance within 10 days of SRC, [2] but the proportions are lower in the non-athlete population (approximately 50%) [57]. Future studies should include a subset of concussed participants who are not experiencing symptom-limited exercise intolerance to understand differences that are specific to those with exercise intolerance. Another limitation is that we used only one modality of exercise (recumbent cycling) to assess exercise intolerance. Recumbent cycling may trigger fewer concussion symptoms as there is less motion of the head and torso; we are unsure if upright cycling or walking on a treadmill would have similar results. Sports-specific exercise that includes focusing on an object in motion (i.e., a ball) or maintaining balance may induce additional symptoms relating to vision and balance. Participants were instructed that they could do light physical activity prior to the first study visit. While we did not formally measure physical activity in the days prior to the first visit, participants had study testing the day they were diagnosed or within 2–3 days of diagnosis. We are confident that permission to walk or perform light household chores did not affect our results, but future studies could consider measuring levels of activity prior to physiological testing. Additionally, cardiopulmonary measurements can be affected by mood, diurnal variation, and baseline levels of stress and fatigue. We did not control for these variables, and we assume any effects to be random, but future studies should attempt to control for them. A history of concussion is another possible confounder. Autonomic physiological abnormalities can be detectable over a year after clinical recovery [58]. Future studies should aim for an equal distribution of participants with a history of concussion. We studied only exercise-intolerant adolescents within 10 days of SRC who participated in sport and recreational activities, so our findings may not apply to younger or older patients with concussion, to the non-athletic population, to the elite athlete population, to those with non-sport mechanisms of injury, to non-exercise-intolerant patients, to those with persisting symptoms, or to those beyond 10 days from injury. Finally, we were unable to test the athletes prior to their injury, so we are unsure if these responses may have been present prior to the concussive head injury.

## Conclusions

This case–control study found that concussed adolescents exercised less because of a concussion symptom exacerbation, reported a higher perceived exertion at similar exercise intensities, and their BP plateaued at lower values when compared with healthy control athletes during equivalent intensities of progressive cycle ergometer exercise. No differences in cardiovascular or respiratory physiology variables at rest or evidence of aerobic deconditioning were found. We speculate that the attenuated BP responses to exercise might have reduced blood flow to the muscles that limited aerobic exercise performance in concussed adolescent athletes. We hypothesize that this is because of the ANS’s inability to respond to increasing physiological demand rather than to cardiac myocyte dysfunction, but this warrants additional research. Novel approaches to restoring normal cardiovascular function, such as targeted HR aerobic exercise, should be investigated to facilitate cardiopulmonary physiological recovery from SRC.

## Supplementary Information

Below is the link to the electronic supplementary material.

Supplementary file1 (DOCX 791 KB)

## Declarations

### Availability of Data and Material

Data can be requested from the corresponding author at haider@buffalo.edu.

## References

[1] Leddy JJ, Baker JG, Kozlowski K, Bisson L, Willer B. Reliability of a graded exercise test for assessing recovery from concussion. Clin J Sport Med. 2011;21(2):89–94. 21358497 10.1097/JSM.0b013e3181fdc721

[2] Haider MN, Lutnick E, Nazir MS, Nowak A, Chizuk HM, Miecznikowski JC, et al. Sensitivity and specificity of exercise intolerance on graded exertion testing for diagnosing sport-related concussion: a systematic review and exploratory meta-analysis. J Neurotrauma. 2023;40(15–16):1524–32. 37014078 10.1089/neu.2022.0331

[3] Leddy JJ, Willer B. Use of graded exercise testing in concussion and return-to-activity management. Curr Sports Med Rep. 2013;12(6):370–6. 24225521 10.1249/JSR.0000000000000008

[4] Haider MN, Leddy JJ, Wilber CG, Viera KB, Bezherano I, Wilkins KJ, et al. The predictive capacity of the Buffalo concussion treadmill test after sport-related concussion in adolescents. Front Neurol. 2019;10:395. 31105634 10.3389/fneur.2019.00395 PMC6492460

[5] Darling SR, Leddy JJ, Baker JG, Williams AJ, Surace A, Miecznikowski JC, et al. Evaluation of the Zurich guidelines and exercise testing for return to play in adolescents following concussion. Clin J Sport Med. 2014;24(2):128–33. 24184849 10.1097/JSM.0000000000000026

[6] Leddy J, Cox J, Baker J, Wack D, Willer B. Exercise treatment for post concussion syndrome: a placebo controlled pilot study of changes in fMRIBlood flow and symptoms. Brain Inj. 2012;26(4–5):388–9.

[7] Leddy JJ, Kozlowski K, Donnelly JP, Pendergast DR, Epstein LH, Willer B. A preliminary study of subsymptom threshold exercise training for refractory post-concussion syndrome. Clin J Sport Med. 2010;20(1):21–7. 20051730 10.1097/JSM.0b013e3181c6c22c

[8] Fu Q, Levine BD. Autonomic nervous system: Chapter 13. Exercise and the autonomic nervous system. Amsterdam, Netherlands: Elsevier Inc.; 2013.

[9] Goldstein B, Toweill D, Lai S, Sonnenthal K, Kimberly B. Uncoupling of the autonomic and cardiovascular systems in acute brain injury. Am J Physiol. 1998;275(4):R1287–92. 9756562 10.1152/ajpregu.1998.275.4.R1287

[10] King M, Lichtman S, Seliger G, Ehert F, Steinberg J. Heart-rate variability in chronic traumatic brain injury. Brain Inj. 1997;11(6):445–53. 9171929 10.1080/026990597123421

[11] Hanna-Pladdy B, Berry ZM, Bennett T, Phillips HL, Gouvier WD. Stress as a diagnostic challenge for postconcussive symptoms: sequelae of mild traumatic brain injury or physiological stress response. Clin Neuropsychol. 2001;15(3):289–304. 11778766 10.1076/clin.15.3.289.10272

[12] Gall B, Parkhouse W, Goodman D. Exercise following a sport induced concussion. Br J Sports Med. 2004;38(6):773–7. 15562179 10.1136/bjsm.2003.009530 PMC1724990

[13] Hinds A, Leddy J, Freitas M, Czuczman N, Willer B. The effect of exertion on heart rate and rating of perceived exertion in acutely concussed individuals. J Neurol Neurophysiol. 2016;7(4): 388. 27812398 10.4172/2155-9562.1000388 PMC5089811

[14] Bruss ZS, Raja A. Physiology, stroke volume. Treasure Island, FL: StatPearls Publishing; 2021. 31613466

[15] Carol MP, Sheila G. Porth’s pathophysiology: concepts of altered health states. 9th ed. Philadelphia, PA: Wolters Kluwer; 2013.

[16] Mossberg KA, Ayala D, Baker T, Heard J, Masel B. Aerobic capacity after traumatic brain injury: comparison with a nondisabled cohort. Arch Phys Med Rehabil. 2007;88(3):315–20. 17321823 10.1016/j.apmr.2006.12.006

[17] Kay JJM, Moore RD. Determining aerobic capacity at symptomatic threshold in individuals with persistent concussion symptoms. Neurology. 2018;91(23):S21–2.29987132

[18] Haider MN, Johnson SL, Mannix R, Macfarlane AJ, Constantino D, Johnson BD, et al. The buffalo concussion bike test for concussion assessment in adolescents. Sports Health. 2019;11(6):492–7. 31486715 10.1177/1941738119870189 PMC6822206

[19] McCrory P, Meeuwisse W, Dvorak J, Aubry M, Bailes J, Broglio S, et al. Consensus statement on concussion in sport: the 5th International Conference on Concussion in Sport held in Berlin, October 2016. Br J Sports Med. 2017;51(11):838–47. 28446457 10.1136/bjsports-2017-097699

[20] Elbin RJ, Knox J, Kegel N, Schatz P, Lowder HB, French J, et al. Assessing symptoms in adolescents following sport-related concussion: a comparison of four different approaches. Appl Neuropsychol Child. 2016;5(4):294–302. 27105069 10.1080/21622965.2015.1077334

[21] Lovell MR, Iverson GL, Collins MW, Podell K, Johnston KM, Pardini D, et al. Measurement of symptoms following sports-related concussion: reliability and normative data for the post-concussion scale. Appl Neuropsychol. 2006;13(3):166–74. 17361669 10.1207/s15324826an1303_4

[22] Selim M, Jones R, Novak P, Zhao P, Novak V. The effects of body mass index on cerebral blood flow velocity. Clin Auton Res. 2008;18(6):331. 18726054 10.1007/s10286-008-0490-z PMC2600600

[23] Riebe D, Ehrman JK, Liguori G, Magal M. Medicine ACoS. ACSM’s guidelines for exercise testing and prescription. Philadelphia, PA: Wolters Kluwer; 2018.

[24] Wesseling K, Jansen J, Settels J, Schreuder J. Computation of aortic flow from pressure in humans using a nonlinear, three-element model. J Appl Physiol. 1993;74(5):2566–73. 8335593 10.1152/jappl.1993.74.5.2566

[25] Eeftinck Schattenkerk DW, Van Lieshout JJ, Van Den Meiracker AH, Wesseling KR, Blanc S, Wieling W, et al. Nexfin noninvasive continuous blood pressure validated against Riva-rocci/Korotkoff. Am J Hypertens. 2009;22(4):378–83. 19180062 10.1038/ajh.2008.368

[26] Lang S, Herold R, Kraft A, Harth V, Preisser AM. Spiroergometric measurements under increased inspiratory oxygen concentration (FIO2): putting the Haldane transformation to the test. PLoS ONE. 2018;13(12): e0207648. 30540773 10.1371/journal.pone.0207648 PMC6291083

[27] Stehlik-Barry K, Babinec AJ. Data analysis with IBM SPSS statistics. Birmingham, UK: Packt Publishing Ltd; 2017.

[28] Gonzalez-Alonso J, Calbet JA. Reductions in systemic and skeletal muscle blood flow and oxygen delivery limit maximal aerobic capacity in humans. Circulation. 2003;107(6):824–30. 12591751 10.1161/01.cir.0000049746.29175.3f

[29] Mortensen SP, Dawson EA, Yoshiga CC, Dalsgaard MK, Damsgaard R, Secher NH, et al. Limitations to systemic and locomotor limb muscle oxygen delivery and uptake during maximal exercise in humans. J Physiol. 2005;566(Pt 1):273–85. 15860533 10.1113/jphysiol.2005.086025 PMC1464731

[30] Lin K, Wei L, Huang Z, Zeng Q. Combination of Ewing test, heart rate variability, and heart rate turbulence analysis for early diagnosis of diabetic cardiac autonomic neuropathy. Medicine (Baltimore). 2017;96(45): e8296. 29137013 10.1097/MD.0000000000008296 PMC5690706

[31] Pertab JL, Merkley TL, Cramond AJ, Cramond K, Paxton H, Wu T. Concussion and the autonomic nervous system: an introduction to the field and the results of a systematic review. NeuroRehabilitation. 2018;42(4):397–427. 29660949 10.3233/NRE-172298 PMC6027940

[32] Johnson BD, O’Leary MC, McBryde M, Sackett JR, Schlader ZJ, Leddy JJ. Face cooling exposes cardiac parasympathetic and sympathetic dysfunction in recently concussed college athletes. Physiol Rep. 2018;6(9): e13694. 29741235 10.14814/phy2.13694 PMC5941219

[33] Johnson BD, Sackett JR, Schlader ZJ, Leddy JJ. Attenuated cardiovascular responses to the cold pressor test in concussed collegiate athletes. J Athl Train. 2020;55(2):124–31. 31909640 10.4085/1062-6050-573-18 PMC7017893

[34] Leddy JJ, Haider MN, Ellis MJ, Mannix R, Darling SR, Freitas MS, et al. Early subthreshold aerobic exercise for sport-related concussion: a randomized clinical trial. JAMA Pediatr. 2019;173(4):319–25. 30715132 10.1001/jamapediatrics.2018.4397 PMC6450274

[35] Leddy JJ, Master CL, Mannix R, Wiebe DJ, Grady MF, Meehan WP, et al. Early targeted heart rate aerobic exercise versus placebo stretching for sport-related concussion in adolescents: a randomised controlled trial. Lancet Child Adolesc Health. 2021;5(11):792–9. 34600629 10.1016/S2352-4642(21)00267-4

[36] Liu S, Ye M, Pao GM, Song SM, Jhang J, Jiang H, et al. Divergent brainstem opioidergic pathways that coordinate breathing with pain and emotions. Neuron. 2022;110(5):857–73.e9. 34921781 10.1016/j.neuron.2021.11.029 PMC8897232

[37] MacKay CM, Skow RJ, Tymko MM, Boulet LM, Davenport MH, Steinback CD, et al. Central respiratory chemosensitivity and cerebrovascular CO_2_ reactivity: a rebreathing demonstration illustrating integrative human physiology. Adv Physiol Educ. 2016;40(1):79–92. 26873894 10.1152/advan.00048.2015

[38] Gargaglioni LH, Hartzler LK, Putnam RW. The locus coeruleus and central chemosensitivity. Respir Physiol Neurobiol. 2010;173(3):264–73. 20435170 10.1016/j.resp.2010.04.024 PMC2929404

[39] Clausen M, Pendergast DR, Wilier B, Leddy J. Cerebral blood flow during treadmill exercise is a marker of physiological postconcussion syndrome in female athletes. J Head Trauma Rehabil. 2016;31(3):215–24. 26098254 10.1097/HTR.0000000000000145

[40] Ballantyne D, Scheid P. Central chemosensitivity of respiration: a brief overview. Respir Physiol. 2001;129(1–2):5–12. 11738642 10.1016/s0034-5687(01)00297-3

[41] Zuj KA, Arbeille P, Shoemaker JK, Blaber AP, Greaves DK, Xu D, et al. Impaired cerebrovascular autoregulation and reduced CO_2_ reactivity after long duration spaceflight. Am J Physiol Heart Circ Physiol. 2012;302(12):H2592–8. 22492717 10.1152/ajpheart.00029.2012

[42] Hart DA. Learning from human responses to deconditioning environments: improved understanding of the “Use It or Lose It” principle. Front Sports Act Living. 2021;3: 68584. 10.3389/fspor.2021.685845 PMC8677937 34927066

[43] Moyna N, Thompson P. The effect of physical activity on endothelial function in man. Acta Physiol Scand. 2004;180(2):113–23. 14738470 10.1111/j.0001-6772.2003.01253.x

[44] Cullinane EM, Sady SP, Vadeboncoeur L, Burke M, Thompson PD. Cardiac size and *V*O_2max_ do not decrease after short-term exercise cessation. Med Sci Sports Exerc. 1986;18(4):420–4. 3747802

[45] Mujika I, Padilla S. Detraining: loss of training-induced physiological and performance adaptations. Part II: long term insufficient training stimulus. Sports Med. 2000;30(3):145–54. 10999420 10.2165/00007256-200030030-00001

[46] Zheng J, Pan T, Jiang Y, Shen Y. Effects of short-and long-term detraining on maximal oxygen uptake in athletes: a systematic review and meta-analysis. BioMed Res Int. 2022;2022:2130993. 36017396 10.1155/2022/2130993 PMC9398774

[47] Levine BD, Zuckerman JH, Pawelczyk JA. Cardiac atrophy after bed-rest deconditioning: a nonneural mechanism for orthostatic intolerance. Circulation. 1997;96(2):517–25. 9244220 10.1161/01.cir.96.2.517

[48] Leddy JJ, Burma JS, Toomey CM, Hayden A, Davis GA, Babl FE, et al. Rest and exercise early after sport-related concussion: a systematic review and meta-analysis. Br J Sports Med. 2023;57(12):762–70. 37316185 10.1136/bjsports-2022-106676

[49] Tan CO, Meehan WP, Iverson GL, Taylor JA. Cerebrovascular regulation, exercise, and mild traumatic brain injury. Neurology. 2014;83(18):1665–72. 25274845 10.1212/WNL.0000000000000944 PMC4223082

[50] Worley M, O’Leary M, Sackett J, Schlader Z, Leddy J, Johnson B. Static cerebral autoregulation is not altered in symptomatic concussed athletes during acute central hypervolemia. Int J Exerc Sci. 2019;2019:124.

[51] Clausen M, Pendergast DR, Willer B, Leddy J. Cerebral blood flow during treadmill exercise is a marker of physiological postconcussion syndrome in female athletes. J Head Trauma Rehabil. 2016;31(3):215–24. 26098254 10.1097/HTR.0000000000000145

[52] Howell DR, Hunt DL, Aaron SE, Hamner JW, Meehan WP 3rd, Tan CO. Association of hemodynamic and cerebrovascular responses to exercise with symptom severity in adolescents and young adults with concussion. Neurology. 2021;97(22):e2204–12. 34635563 10.1212/WNL.0000000000012929 PMC8641971

[53] Tan CO, Hamner J, Taylor JA. The role of myogenic mechanisms in human cerebrovascular regulation. J Physiol. 2013;591(20):5095–105. 23959681 10.1113/jphysiol.2013.259747 PMC3810812

[54] Callaway CCM, Kosofsky BE. Autonomic dysfunction following mild traumatic brain injury. Curr Opin Neurol. 2019;32(6):802–7. 31567549 10.1097/WCO.0000000000000751

[55] Goodman B, Vargas B, Dodick D. Autonomic nervous system dysfunction in concussion. Neurology. 2013;80(7_supplement) IN5-2.005.

[56] Polak P, Leddy JJ, Dwyer MG, Willer B, Zivadinov R. Diffusion tensor imaging alterations in patients with postconcussion syndrome undergoing exercise treatment: a pilot longitudinal study. J Head Trauma Rehabil. 2015;30(2):E32–42. 24721808 10.1097/HTR.0000000000000037

[57] Orr R, Bogg T, Fyffe A, Lam LT, Browne GJ. Graded exercise testing predicts recovery trajectory of concussion in children and adolescents. Clin J Sport Med. 2021;31(1):23–30. 30439726 10.1097/JSM.0000000000000683

[58] Haider MN, Johnson BD, Horn EC, Leddy JJ, Wilber CG, Reed EL, et al. Blunted cardiac parasympathetic activation in student athletes with a remote history of concussion: a pilot study. Front Neurol. 2020;11:1156. 10.3389/fneur.2020.547126 PMC7554519 33101172

