# Supplementary material for: Cardiovascular and Respiratory Responses During Graded Exercise in Adolescents After Sport-Related Concussion
Source: Sports Med. 2025 Aug 26;56(1):271–82. doi: 10.1007/s40279-025-02301-7 (PMC12913255; doi:10.1007/s40279-025-02301-7)
Supplement: Supplementary file 1 — Supplementary file1 (DOCX 791 KB) [file 40279_2025_2301_MOESM1_ESM.docx]

**Supplementary File for “Cardiovascular and Respiratory Responses During Graded Exercise in Adolescents after Sport-Related Concussion”**

This supplement file presents additional analysis of cardiovascular and respiratory exercise in a sample of concussed adolescents and matched controls. The hypothesis is that there will be some evidence of limitations to exercise acutely after injury. All statistical analysis is performed on SPSS Version 29 and graphs are made on Prism GraphPad Version 10.

Contents

[Missing data 1](#_Toc153984364)

[**Figure e1.** Sample inclusion flow chart 2](#_Toc153984365)

[**Table e1.** List of sports played by groups 3](#_Toc153984366)

[**Table e2.** Cardiovascular variables 3](#_Toc153984367)

[**Figure e2.** Cardiovascular variables plotted against time on BCBT 6](#_Toc153984368)

[**Table e3.** Respiratory variables 7](#_Toc153984369)

[**Figure e3.** Respiratory variables plotted against time on BCBT 8](#_Toc153984370)

#### Missing data

Concussed adolescents went for a mean of 8.12 stages (16.24 minutes), however, the percentage of missing data up to Stage 9 of the BCBT is around 34%. Apart from some missing variables in between, majority of missing data is at Stage 6 and beyond when the acutely concussed group ended the test due to symptom exacerbation. Unlike a repeated measures model, a generalized linear model can handle missing data. However, any results we obtain with this much missing data will be considered hypothesis generating as opposed to hypothesis testing.^1^ Hence, we will only compare groups up to Stage 5 which is 10 minutes of exercise on the BCBT since all concussed participants had reached this stage and there was minimal missing data.

1. Clark TG, Altman DG. Developing a prognostic model in the presence of missing data: an ovarian cancer case study. J Clin Epidemiol. 2003;56(1):28–37.

## **Figure e1.** Sample inclusion flow chart


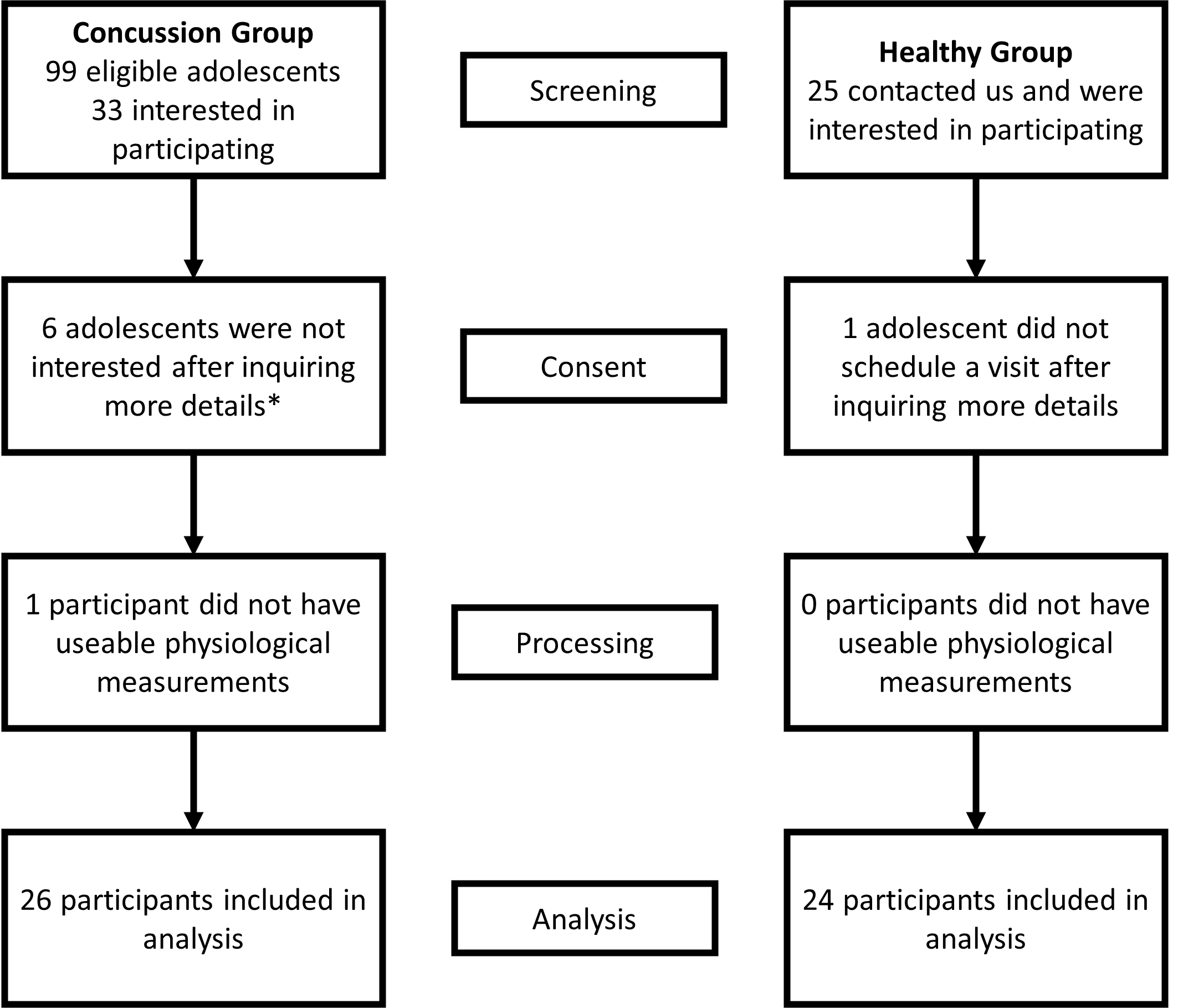


*all participants/parents said they were unable to schedule a 4-hour long research visit within a couple of days of first doctor’s appointment

## **Table e1.** Cardiovascular variables

|  | **Concussion Group** | | **Healthy Group** | |
| --- | --- | --- | --- | --- |
|  | **Mean** | **Standard Deviation** | **Mean** | **Standard Deviation** |
| **Cardiac Output (L/minute)** | | | | |
| Baseline | 7.28 | 1.21 | 7.19 | 1.47 |
| Stage 1 | 11.03 | 1.75 | 10.34 | 2.00 |
| Stage 2 | 11.45 | 1.83 | 11.12 | 2.00 |
| Stage 3 | 12.13 | 1.83 | 12.22 | 2.25 |
| Stage 4 | 12.64 | 2.01 | 13.02 | 2.17 |
| Stage 5 | 13.43 | 2.22 | 13.95 | 2.20 |
| **Diastolic Blood Pressure (mmHg)** | | | | |
| Baseline | 72.72 | 11.02 | 74.64 | 6.96 |
| Stage 1 | 75.10 | 8.89 | 77.62 | 7.79 |
| Stage 2 | 77.42 | 8.98 | 79.30 | 6.77 |
| Stage 3 | 78.43 | 9.20 | 81.47 | 6.54 |
| Stage 4 | 77.90 | 10.35 | 81.96 | 7.25 |
| Stage 5 | 76.53 | 10.02 | 81.83 | 7.49 |
| **Heart Rate (beats/minute)** | | | | |
| Baseline | 75.36 | 9.00 | 73.85 | 9.73 |
| Stage 1 | 99.30 | 13.75 | 95.70 | 11.13 |
| Stage 2 | 100.86 | 12.92 | 101.39 | 11.93 |
| Stage 3 | 106.70 | 10.67 | 108.86 | 14.58 |
| Stage 4 | 115.40 | 12.92 | 112.66 | 12.10 |
| Stage 5 | 116.66 | 10.79 | 119.30 | 12.80 |
| **Mean Arterial Pressure (mmHg)** | | | | |
| Baseline | 94.05 | 11.03 | 95.79 | 7.638 |
| Stage 1 | 99.12 | 10.96 | 100.67 | 9.585 |
| Stage 2 | 101.78 | 10.67 | 102.52 | 9.124 |
| Stage 3 | 102.77 | 10.78 | 105.82 | 8.903 |
| Stage 4 | 101.64 | 12.62 | 106.56 | 9.494 |
| Stage 5 | 100.77 | 12.05 | 107.05 | 9.435 |
| **Systolic Blood Pressure (mmHg)** | | | | |
| Baseline | 124.03 | 15.34 | 124.08 | 11.83 |
| Stage 1 | 135.89 | 14.79 | 133.16 | 15.13 |
| Stage 2 | 139.73 | 14.55 | 135.96 | 14.65 |
| Stage 3 | 141.62 | 14.86 | 141.11 | 13.88 |
| Stage 4 | 140.35 | 17.41 | 143.82 | 13.99 |
| Stage 5 | 140.20 | 18.26 | 146.25 | 14.06 |
| **Stroke Volume (cm^3^)** | | | | |
| Baseline | 97.33 | 13.85 | 98.38 | 19.56 |
| Stage 1 | 114.88 | 16.28 | 109.61 | 22.81 |
| Stage 2 | 115.93 | 17.10 | 110.53 | 22.60 |
| Stage 3 | 115.71 | 17.02 | 113.43 | 23.49 |
| Stage 4 | 111.66 | 17.74 | 115.11 | 22.68 |
| Stage 5 | 114.24 | 18.27 | 116.87 | 22.09 |
| **Systemic Vascular Resistance** | | | | |
| Baseline | 13.13 | 1.80 | 13.83 | 2.77 |
| Stage 1 | 9.19 | 1.80 | 10.07 | 2.02 |
| Stage 2 | 9.09 | 1.69 | 9.50 | 1.83 |
| Stage 3 | 8.65 | 1.58 | 8.94 | 1.71 |
| Stage 4 | 8.26 | 1.87 | 8.42 | 1.64 |
| Stage 5 | 7.75 | 2.10 | 7.86 | 1.38 |

**Figure e2.** Cardiovascular variables plotted against time on BCBT
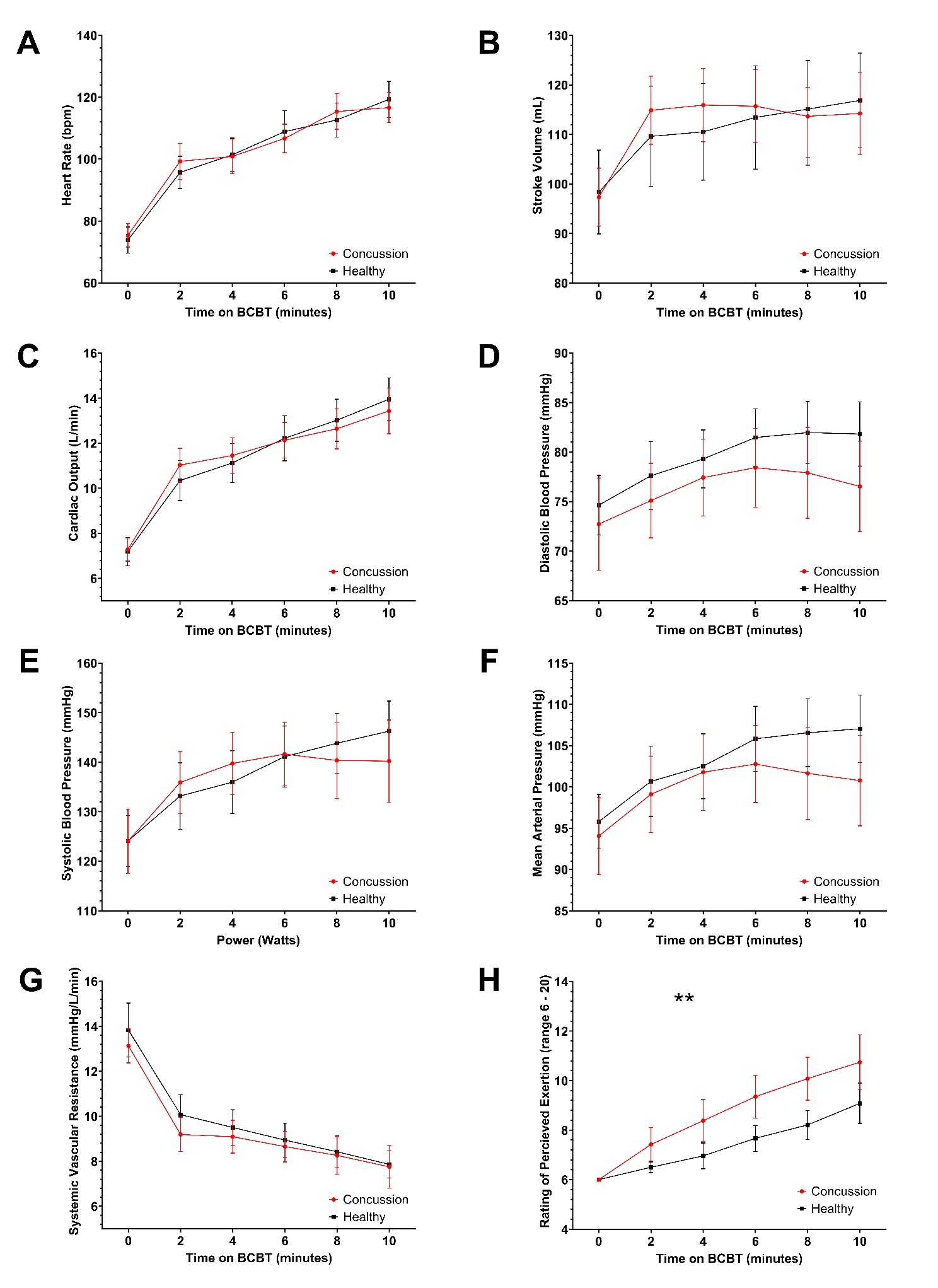


## **Table e2.** Respiratory variables

|  | **Concussion Group** | | **Healthy Group** | |
| --- | --- | --- | --- | --- |
|  | Mean | Standard Deviation | Mean | Standard Deviation |
| **Minute Ventilation (cm^3^/minute)** | | | | |
| Baseline | 8325 | 2035 | 8287 | 2207 |
| Stage 1 | 16245 | 4228 | 15172 | 3275 |
| Stage 2 | 18684 | 4334 | 17576 | 3570 |
| Stage 3 | 21312 | 4446 | 19447 | 4080 |
| Stage 4 | 23391 | 5239 | 21508 | 4972 |
| Stage 5 | 25665 | 6230 | 24250 | 5500 |
| **FeCO_2_** | | | | |
| Baseline | 0.016 | 0.0083 | 0.015 | 0.0054 |
| Stage 1 | 0.025 | 0.0075 | 0.026 | 0.0064 |
| Stage 2 | 0.029 | 0.0077 | 0.030 | 0.0051 |
| Stage 3 | 0.031 | 0.0074 | 0.033 | 0.0055 |
| Stage 4 | 0.031 | 0.0068 | 0.034 | 0.0057 |
| Stage 5 | 0.032 | 0.0065 | 0.035 | 0.0061 |
| **FeO_2_** | | | | |
| Baseline | 0.18 | 0.0075 | 0.18 | 0.0052 |
| Stage 1 | 0.17 | 0.0059 | 0.17 | 0.0054 |
| Stage 2 | 0.16 | 0.0063 | 0.17 | 0.0040 |
| Stage 3 | 0.16 | 0.0056 | 0.17 | 0.0039 |
| Stage 4 | 0.17 | 0.0046 | 0.17 | 0.0040 |
| Stage 5 | 0.17 | 0.0044 | 0.17 | 0.0047 |
| **Respiratory Rate (breaths/minute)** | | | | |
| Baseline | 18.94 | 2.99 | 18.20 | 5.25 |
| Stage 1 | 22.79 | 5.85 | 21.97 | 3.23 |
| Stage 2 | 24.73 | 4.65 | 22.59 | 3.78 |
| Stage 3 | 26.01 | 4.92 | 23.10 | 3.88 |
| Stage 4 | 26.88 | 5.16 | 23.83 | 4.77 |
| Stage 5 | 28.16 | 4.67 | 25.11 | 5.16 |
| **Tidal Volume (cm^3^)** | | | | |
| Baseline | 431.82 | 142.47 | 450.23 | 111.34 |
| Stage 1 | 664.32 | 182.79 | 709.40 | 164.03 |
| Stage 2 | 739.96 | 190.10 | 788.27 | 187.58 |
| Stage 3 | 813.49 | 252.71 | 848.09 | 194.48 |
| Stage 4 | 888.66 | 222.75 | 910.91 | 197.30 |
| Stage 5 | 915.04 | 191.23 | 978.20 | 196.22 |
| **VO_2_** | | | | |
| Baseline | 3.57 | 1.16 | 3.64 | 1.29 |
| Stage 1 | 9.04 | 1.58 | 8.81 | 1.92 |
| Stage 2 | 10.17 | 1.64 | 10.47 | 1.74 |
| Stage 3 | 11.83 | 1.86 | 11.73 | 1.64 |
| Stage 4 | 12.81 | 1.80 | 12.86 | 1.69 |
| Stage 5 | 13.84 | 1.61 | 14.38 | 1.56 |
| **End Tidal CO_2_ (mmHg)** | | | | |
| Baseline | 35.144 | 4.411 | 32.066 | 7.189 |
| Stage 1 | 38.627 | 4.283 | 37.921 | 5.572 |
| Stage 2 | 39.371 | 4.318 | 38.277 | 7.030 |
| Stage 3 | 39.590 | 4.095 | 40.151 | 6.335 |
| Stage 4 | 40.033 | 4.147 | 39.859 | 6.906 |
| Stage 5 | 39.711 | 4.319 | 39.991 | 7.053 |

## **Figure e3.** Respiratory variables plotted against time on BCBT

**
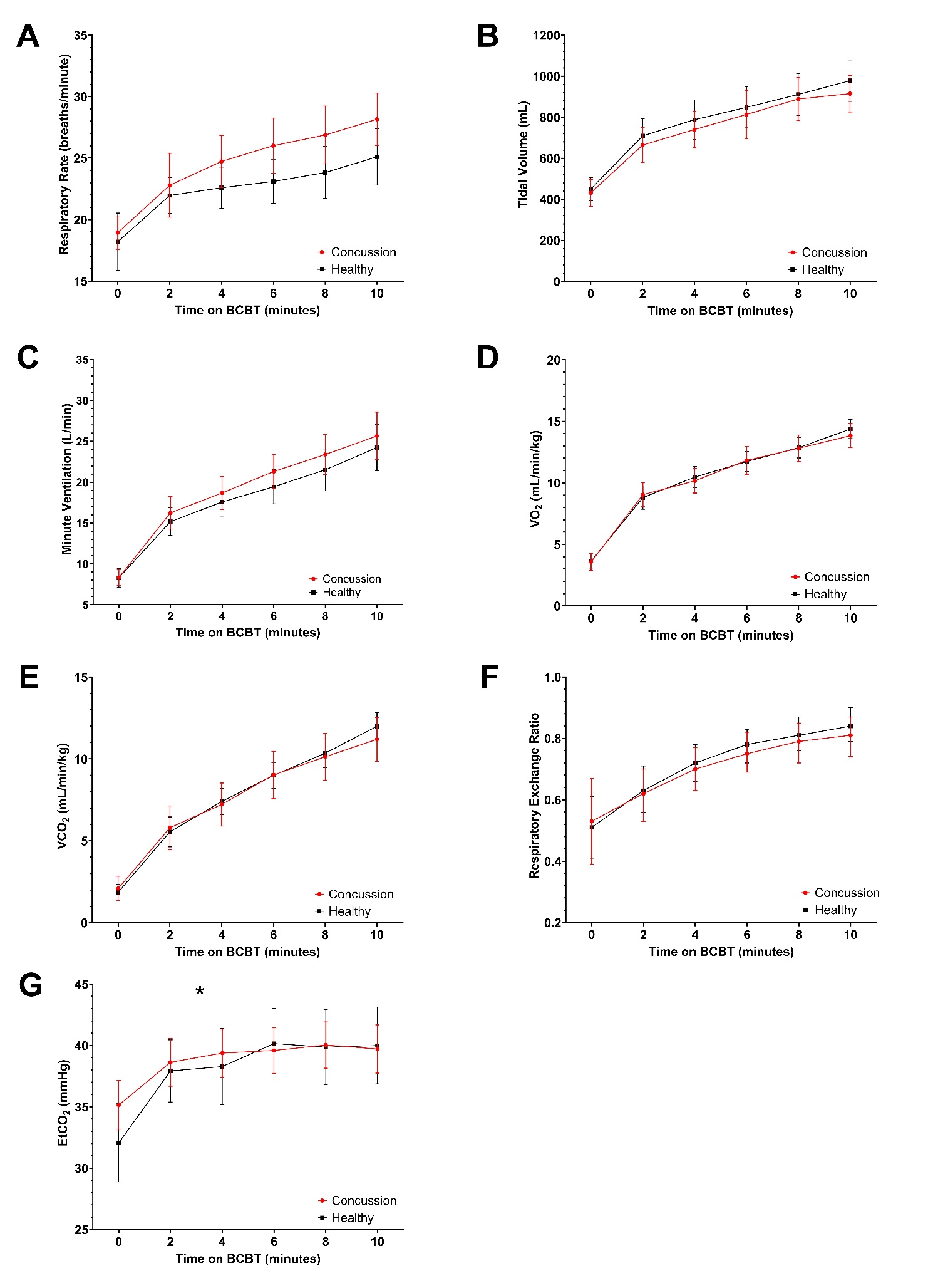
**

**Table e3.** Groupwise cardiovascular and respiratory responses during the final minute of exercise

|  | **Concussion Group** | **Healthy Group** | ***p*-value** |
| --- | --- | --- | --- |
| Rating of Perceived Exertion | 14.81 ± 3.24 | 17.42 ± 1.95 | **0.001** |
| Power output (watts) | 112.26 ± 30.80 | 150.12 ± 32.08 | **< 0.001** |
| **Cardiovascular** | | | |
| Heart Rate (beats/minute) | 138.03 ± 30.56 | 165.35 ± 18.18 | **< 0.001** |
| Stroke Volume (mL) | 111.10 ± 23.69 | 115.36 ± 22.47 | 0.531 |
| Cardiac Output (L/minute) | 15.18 ± 3.79 | 17.77 ± 2.44 | **0.008** |
| Diastolic Blood Pressure (mmHg) | 78.57 ± 10.20 | 82.24 ± 9.58 | 0.211 |
| Systolic Blood Pressure (mmHg) | 141.40 ± 20.11 | 148.54 ± 19.27 | 0.221 |
| Mean Arterial Pressure (mmHg) | 105.35 ± 12.25 | 112.47 ± 15.14 | 0.082 |
| Systemic Vascular Resistance (mmHg/L/min) | 7.75 ± 4.25 | 6.55 ± 1.99 | 0.222 |
| **Respiratory** | | | |
| Respiratory Rate (breaths/minute) | 35.12 ± 8.17 | 38.59 ± 8.37 | 0.167 |
| Tidal Volume (mL) | 1094.26 ± 324.79 | 1259.99 ± 292.70 | 0.105 |
| Minute Ventilation (L/minute) | 39.23 ± 12.04 | 48.66 ± 15.46 | **0.039** |
| VO_2_ (mL/min/kg) | 20.02 ± 4.28 | 25.58 ± 7.90 | **0.029** |
| VCO_2_ (mL/min/kg) | 17.45 ± 4.68 | 23.06 ± 8.08 | **0.022** |
| RER (ratio) | 0.87 ± 0.11 | 0.89 ± 0.12 | 0.543 |
| EtCO_2_ (mmHg) | 37.20 ± 4.88 | 35.07 ± 6.63 | 0.230 |

*Bolded values indicate a significant finding; EtCO_2_: end-tidal CO_2_; VO_2_: oxygen consumption; VCO_2_: carbon dioxide consumption; RER: respiratory exchange ratio.*

**Table e4.** Medians and interquartile range for Table 2

|  | **Concussion** | | | **Healthy** | | |
| --- | --- | --- | --- | --- | --- | --- |
|  | Median | Percentile 25 | Percentile 75 | Median | Percentile 25 | Percentile 75 |
| HR | 75.94 | 69.08 | 80.07 | 72.88 | 67.93 | 79.91 |
| SV | 94.97 | 88.99 | 106.28 | 99.46 | 86.55 | 113.45 |
| CO | 7.04 | 6.40 | 8.15 | 6.94 | 6.28 | 7.97 |
| DBP | 72.06 | 69.16 | 74.56 | 74.23 | 69.95 | 81.44 |
| SBP | 120.25 | 115.12 | 127.49 | 126.79 | 112.63 | 132.59 |
| MAP | 93.32 | 89.17 | 95.89 | 95.16 | 90.44 | 102.26 |
| SVR | 13.47 | 11.91 | 14.34 | 13.89 | 12.85 | 15.14 |
| RR | 17.84 | 16.99 | 20.37 | 19.05 | 16.77 | 20.67 |
| TV | 468.06 | 329.36 | 550.53 | 458.68 | 387.63 | 495.95 |
| MV_in_L | 8.67 | 7.23 | 9.93 | 8.34 | 7.08 | 9.30 |
| VO2 | 4.00 | 2.63 | 4.318 | 4.032 | 2.90 | 4.55 |
| VCO2mlminkg | 2.01 | 1.23 | 3.05 | 1.88 | 1.19 | 2.46 |
| RER | 0.54 | 0.40 | 0.64 | 0.50 | 0.44 | 0.61 |
| EtCO2 | 33.31 | 32.59 | 38.11 | 34.06 | 31.23 | 35.69 |
